# A fatal *Candida albicans* pericarditis presenting with cardiac tamponade after COVID‐19 infection and cardiothoracic surgery

**DOI:** 10.1002/jcla.24968

**Published:** 2023-10-06

**Authors:** Maryam Salimi, Lotfollah Davoodi, Rozita Jalalian, Masood Darayee, Azam Moslemi, Leyla Faeli, Roghayeh Mirzakhani, Tahereh Shokohi

**Affiliations:** ^1^ Student Research Committee Mazandaran University of Medical Sciences Sari Iran; ^2^ Department of Infectious Diseases, Antimicrobial Resistance Research Center, Communicable Diseases Institute Mazandaran University of Medical Sciences Sari Iran; ^3^ Department of Cardiology, School of Medicine, Cardiovascular Research Center Mazandaran University of Medical Sciences Sari Iran; ^4^ Department of Cardiac Surgery, School of Medicine, Cardiovascular Research Center Mazandaran University of Medical Sciences Sari Iran; ^5^ Mazandaran Heart Center Mazandaran University of Medical Sciences Sari Iran; ^6^ Invasive Fungi Research Center, Communicable Diseases Institute Mazandaran University of Medical Sciences Sari Iran; ^7^ Department of Medical Mycology, School of Medicine Mazandaran University of Medical Sciences Sari Iran

**Keywords:** *Candida*, cardiac tamponade, COVID‐19, diabetes, pericardiocentesis, pericarditis

## Abstract

**Background:**

*Candida* pericardial infection is a rare clinical entity usually related to recent cardiothoracic surgery and chronic debilitating conditions. During the COVID‐19 pandemic, invasive fungal infections have been on the rise, likely due to a combination of factors such as immunosuppression, underlying conditions like diabetes, and surgical procedures.

**Case Presentation:**

Herein, we report a 67‐year‐old diabetic woman with a history of COVID‐19 infection who received a high dose of corticosteroids a few months before admission, and previous myocardial infarction for more than 12 years. The patient had a positive cardiac tamponade with signs of dyspnea, chest pain, and low blood pressure. Echocardiographic data were more in favor of constrictive pericarditis. The patient underwent urgent echocardiography‐guided pericardiocentesis and then broad‐spectrum antibiotic treatment was prescribed. Repeated echocardiography implied a persistent pericardial effusion 10 days later. Subxiphoid aspirates and biopsied tissues showed budding yeast cells and yeast colonies grew on culture media identified as *Candida albicans*.

**Conclusion:**

This report should bring to the attention of physicians toward the possibility of *Candida* pericardial infection presenting with cardiac tamponade after COVID‐19 infection and cardiothoracic surgery. Echocardiographic assessment, prompt pericardiotomy, molecular‐based identification of causative agent, and early administration of appropriate antifungal treatment should improve the patient's survival.

## Graphical abstract

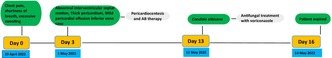

## 1 INTRODUCTION

Pericarditis is an inflammation of the pericardium which can be infectious and non‐infectious. The most common causes of infectious pericarditis are virus, bacteria, and fungi.^1^ Most of them are idiopathic and viral, a benign condition that resolves spontaneously. Pericarditis is a known complication of a number of viral infections, including the flu virus, coxsackievirus, echovirus, and coronavirus.^2^ In a report, 4.6% of patients with COVID‐19 infection had evidence of pericardial effusion.^3^ Purulent pericarditis while rare, is characterized by suppurative pericardial fluid, which usually comes from the expansion of a bacterial infection site near or through the diffusion of blood.^4^ Bacteria and fungi are relatively uncommon cause but more likely lead to purulent pericarditis with cardiac tamponade and pericardial constriction consequences.^4^ Non‐infectious pericarditis following acute myocardial infarction has also been reported.^5^ This condition is often associated with fatal outcomes.

Fungal pericarditis is usually caused by *Candida* species, while *Aspergillus* species have also been reported^4^ that can be fatal if not treated promptly and appropriately.^6^, ^7^ *Candida* pericarditis is a rare condition described primarily in patients with recent cardiothoracic surgery, chronic debilitating illnesses, and immunosuppression resulting from malignancy, chronic therapy with corticosteroids and antibiotics or primarily by gastropericardial fistulas following gastric surgery, which is usually fatal and, unless treated, leads to impaired cardiac function.^8^ *Candida* pericarditis is caused by at least 15 potentially virulent *Candida* species. *Aspergillus* species have been also reported as causative agents in immunocompromised patients.^9^ Amphotericin B and surgical drainage have been reported successful.^7^ Overall mortality due to this clinical entity is significantly high (75%) and is attributed mainly to difficulty in diagnosis due to subtle clinical clues and insidious onset.^10^ Here, we report a fatal case of purulent pericarditis caused by *Candida albicans* presenting with cardiac tamponade after COVID‐19 infection and cardiothoracic surgery.

## 2 CASE REPORT

**FIGURE 1:**
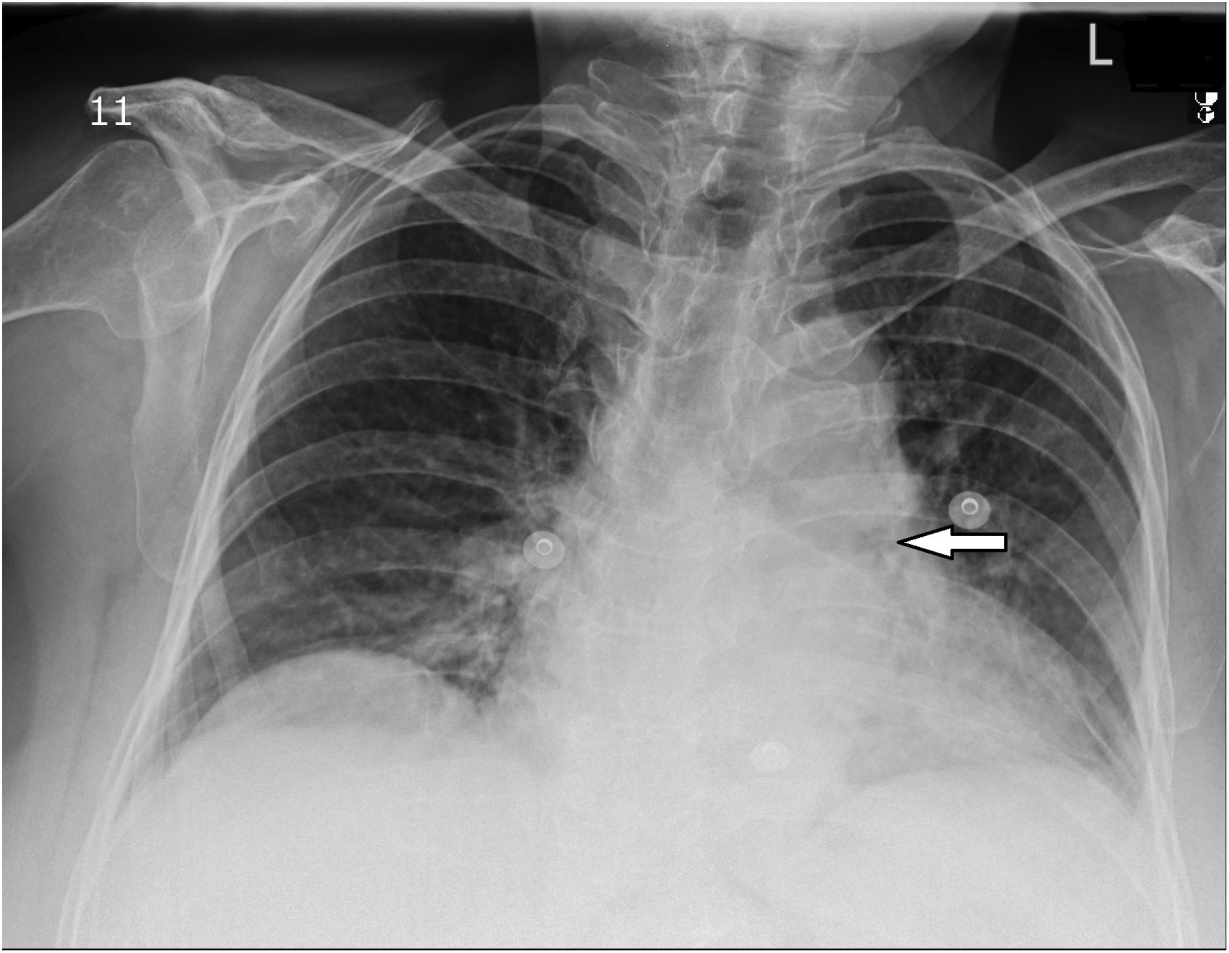
Chest x‐ray showing cardiomegaly.

**FIGURE 2:**
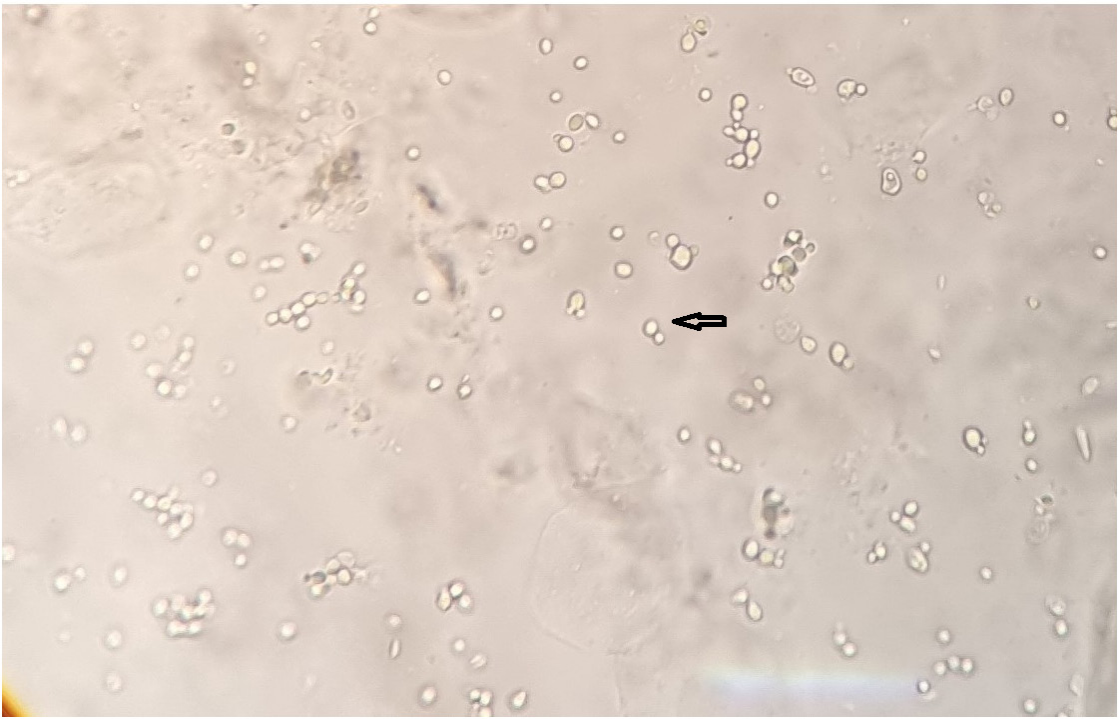
Direct microscopy examination of biopsied tissues and the secretions showing budding yeast cells.

On April 29, 2022, a 67‐year‐old diabetic woman with a past medical history of confirmed SARS‐CoV‐2 infection and receiving a high dose of corticosteroids about 6 months before admission, and previous myocardial infarction (about 12 years before hospitalization), was referred to the Mazandaran Heart Center affiliated with the Mazandaran University of Medical Sciences, northern Iran, complaining of chest pain, shortness of breath, excessive sweating, and pressure in the left thoracic region that had spread to the left hand, decreased in systolic blood pressure, and tachycardia. On May 1, 2022, the laboratory tests showed mild leukocytosis (WBC count: 11,300/μL: including neutrophils 85% and lymphocytes 13%) with elevated levels of inflammation biomarkers such as erythrocyte sedimentation rate (111 mm/h) and C‐reactive protein (77 mg/L). The transthoracic echocardiography has demonstrated left ventricular (LV) hypertrophy with normal LV systolic function, abnormal interventricular septal motion, and septal shift (septal bounce), mild left atrial enlargement, significant respiratory variation mitral valve and tricuspid valve (more than 40%), thick aortic valve with mild aortic insufficiency, a thick pericardium with mild pericardial effusion with dilated inferior vena cava (IVC) without inspiratory collapse. Echocardiographic data were more in favor of constrictive pericarditis consistent with cardiac tamponade physiology; the patient underwent urgent echocardiography‐guided pericardiocentesis and empiric, broad‐spectrum antibiotics including meropenem (1 g IV q8hr) and vancomycin (1 g IV q12hr) were prescribed. The laboratory tests showed that the patient had leukocytosis with a WBC count of 44,300/μL. Additionally, pericardial fluid analysis revealed an average of 14,600 leukocytes/μL, with a differential count predominantly consisting of neutrophils (95%) and lymphocytes (5%). Chest x‐ray showed cardiomegaly, and a mass of 5 cm under the previous surgical scar, which was moved by the pressure of the probe (Figure 1). Repeat echocardiography (on May 10, 2022) results were normal LV size and function (LVEF 50%), moderate LV hypertrophy (LVH), septal hypokinesia, normal right ventricle size and function, thick mitral valve with mitral regurgitation, mild tricuspid regurgitation, mild pericardial effusion, normal pulmonary arterial pressure, with normal sinus rhythm in ECG, which all data implied a persistent pericardial effusion. So, the previous subxiphoid incision was opened, a large amount of pus was removed from the right atrium, and the sample was sent to a laboratory for diagnosis. The microbiological culture of the pericardial fluid yielded *Candida albicans* and antifungal treatment with intravenous voriconazole (loading dose, 400 mg q12 h and maintenance dose, 200 mg q12 h) was started. The direct microscopy examination of biopsied tissues and the secretions showed budding yeast cells (Figure 2), and culture on sabouraud dextrose agar after 1‐day incubation at 37°C yielded moist yeast colonies. Based on microscopic and macroscopic characteristics, it was identified as a *Candida* species and confirmed by polymerase chain reaction (PCR) of the ITS gene and sequencing. The amplicons were sequenced and compared with the GenBank database (https://blast.ncbi.nlm.nih.gov/Blast.cgi) for accurate identification. Based on nucleotide BLAST, the isolate was identified as *C. albicans*. The sequences were submitted to GenBank and assigned the accession number OP268627.1. The in vitro antifungal susceptibility testing (AFST) of the isolate was performed according to the Clinical and Laboratories Standards Institute protocol (CLSI M27‐S4).^11^ In line with the CLSI breakpoint revision, the isolate was resistant to voriconazole (16 μg/mL), itraconazole (16 μg/mL), fluconazole (64 μg/mL), caspofungin (8 μg/mL), micafungin (8 μg/mL), and anidulafungin (1 μg/mL) and sensitive to amphotericin B (0.125 μg/mL) and posaconazole (2 μg/mL). The patient died 3 days after the start of voriconazole treatment. The graphical abstract shows a chronological summary of the patient's manifestation, laboratory diagnosis, and treatment.

## 3 DISCUSSION

*Candida* pericarditis is a rare infection that proceeds to cardiac tamponade and pericardial constriction often with a fatal outcome.^9^ Risk factors include old age, diabetes, and immunosuppression due to malignancy, prolonged steroid therapy, and septicemia. It sometimes occurs after cardiovascular and gastric surgery.^8^ The present case had multiple complex conditions including preexisting cardiac disease; myocardial infarction; immunosuppression resulting from previous infection with COVID‐19 and high‐dose corticosteroid usage, diabetes, and thoracic surgery may be associated with increased risk for pericarditis. We suppose the pericardial effusion has been developed during COVID‐19 infection. Some research suggests that COVID‐19 can cause congestive heart failure, myocardial infarction, and pericardial effusion due to a severe inflammatory process rather than direct invasion of the virus to the myocardium or pericardium.^3^, ^12^ According to previous studies, out of 660 cases of purulent pericarditis, most cases were caused by bacteria, while only 1% were caused by *Candida* species.^13^ *C. albicans* is the most common agent,^14^ followed by *C. tropicalis*, *C. glabrata*, *C. guilliermondii*, *C. parapsilosis*, and *C. auris*.^14^, ^15^, ^16^ More than half of all cases of *Candida* pericarditis were diagnosed after death.^7^ Therefore, a high level of clinical suspicion and early diagnosisare critical to begin the treatment. Immune suppression and recent gastric or cardiovascular surgery are among the most important risk factors for *Candida* pericarditis.^14^

In previous studies, four cases of *Candida* pericarditis were reported with a gastrointestinal source. Two patients were immunocompetent and underwent gastric surgery, and two patients had a history of gastric cancer. *C. albicans* were isolated in two cases, while *C. krusei* and *C. glabrata* were found in other cases.^7^, ^15^, ^17^, ^18^ However, in our case, the source of infection is not clear, it probably has been superinfected by gastrointestinal or skin sources during the first pericardiocentesis. Effusions may become infected by the fungal organism that entered accidentally during the surgical procedure and overgrowth with broad‐spectrum antibiotics or steroids.^19^ One of the most important problems in health care is the development of hospital‐acquired infections, which leads to longer hospital stays and higher mortality.^20^, ^21^ A study of the prevalence of nosocomial infections at a Canadian heart surgery center shows an increase in the prevalence over 18 years from 8% in 1995 to 20% in 2013.^21^ In our patient, we tried to drain the effusion with pericardiocentesis that relieved the tamponed temporarily but due to the entrance to the posterior pericardium and loss of pericardial septa, led to persistent infection and recurrent effusions. We suggest that in case of a positive cardiac tamponade and thick pericardium, complete drainage by pericardiectomy and epicardiectomy is preferable to pericardiocentesis and prevents the recurrence of infection.^13^ The main treatment for *Candida* pericarditis is antifungal treatment and surgical drainage. The most commonly used drug in the studies was amphotericin B, followed by fluconazole.^6^ In a report from a patient with *Candida* pericarditis,^14^ the use of fluconazole and echinocandin was successful, whereas in our case, the isolate was completely resistant to fluconazole and echinocandin.

## 4 CONCLUSION

This report should bring physicians' attention to the possibility of *Candida* pericardial infection presenting with cardiac tamponade after COVID‐19 infection and cardiothoracic surgery. Performing comprehensive echocardiographic assessment, molecular‐based identification of the causative agent, prompt a complete pericardiotomy to ensure no recurrence of the infection, and early administration of appropriate antifungal treatment based on susceptibility test should improve the patient's survival.

## AUTHOR CONTRIBUTIONS

All authors have made substantial contributions to the conception and acquisition of data, drafting of the article, and approval of the final version.

## FUNDING INFORMATION

The authors thank the Invasive Fungi Research Center of Mazandaran University of Medical Sciences, Sari, Iran, for financial support (Grant No. 1327). The funder had no role in the design of this study and will not have any role in the preparation of this manuscript so, no financial interests are available related to the material to disclose.

## CONFLICT OF INTEREST STATEMENT

All authors have no conflicts of interest to declare regarding the publication of the present study.

## Data Availability

The DNA sequences of the studied isolate are available in [NCBI‐ GenBank] at https://www.ncbi.nlm.nih.gov/nuccore/OP268627.1, accession number [OP268627.1].
